# Airway Epithelium in Atopic and Nonatopic Asthma: Similarities and Differences

**DOI:** 10.5402/2011/195846

**Published:** 2011-12-29

**Authors:** Prathap Pillai, Chris J. Corrigan, Sun Ying

**Affiliations:** MRC and Asthma UK Centre for Allergic Mechanisms of Asthma, The Department of Asthma, Allergy and Respiratory Science, Tower Wing, Guy's Hospital, King's College London, London SE1 9RT, UK

## Abstract

Asthma is an inflammatory disorder of the airways, and the airway epithelium has the central role in its pathogenesis. In general, the airway inflammation is characterised by the infiltration of the epithelium and submucosa by a range of inflammatory cells driven largely by Th-2 lymphocytes, eosinophils, and mast cells. The pathogenic mechanisms of nonatopic asthma in comparison to its atopic counterpart have always been a subject of debate. Although clinically are two distinct entities, more similarities than differences have been observed between the two in terms of immunopathogenesis, underlying IgE mechanisms, and so on. in a number of previous studies. More information has become available in recent years comparing the ultrastructure of the epithelium in these two types of asthma. A comparison of airway epithelium in atopic and nonatopic asthma is presented here from the available information in the literature. Similarities outnumber the differences, until we unravel the mystery surrounding these two important phenotypes of asthma in more detail.

## 1. Introduction

Asthma is a common chronic disorder of the airways that is complex and characterized by variable and recurring symptoms, airflow obstruction, bronchial hyper responsiveness, and underlying inflammation [1]. Asthma may be classified clinically on the basis of various parameters including the atopic status of the individual, the degree of airway obstruction, or the nature of trigger factors. By convention, the classification into atopic or nonatopic asthma is based on the presence or absence of clinical symptoms precipitated by one or more common aeroallergens, supported by the presence of allergen-specific antibodies as evidenced by skin prick +/− serological tests. The airways epithelium likely plays a key role in the pathogenesis of asthma, as it is a key interface with the external environment. There is ongoing debate as to whether atopic and nonatopic asthma are immunopathologically two distinct entities or if both are driven by similar mechanisms, and the answer to this question is still not clear. The entity of intrinsic or nonatopic asthma continues to raise questions about the possible role of IgE-mediated mechanisms in asthma pathogenesis. The clarification of this issue becomes more relevant with the current availability of anti-IgE therapy for the treatment of atopic asthma. The main theme of this paper is to address airway epithelium as central to the theatre of asthmatic inflammation and to compare the airways microenvironment in atopic and nonatopic asthma. 

## 2. Airway Epithelium

The airway epithelium is the first site of contact for both inflammatory and physical environmental stimuli. Interaction of epithelial cells with such stimuli may contribute substantially to inflammation and the airways obstruction and oedema seen in asthma and a number of other respiratory diseases. 

Mammalian airways are lined by a variety of specialized cells with critical functions. Eight morphologically distinct epithelial cell types are present in human respiratory epithelium. They are ciliated columnar cells, mucous cells (goblet cells), Clara cells, serous cells, neuroendocrine cells, basal cells, intraepithelial nerves and immune cells. On the basis of ultrastructural, functional, and biochemical criteria, these may be classified into three categories: basal, ciliated, and secretory [2]. Immune cells, inflammatory cells, and phagocytic cells migrate to and remain within the epithelium or transit through to the lumen [3].

### 2.1. Airway Epithelium in Asthma

Asthma is a condition characterised by variable airways obstruction, airways hyper-responsiveness, and chronic airways inflammation. There is increasing evidence suggesting that the airway epithelium has an important role in orchestrating this inflammatory response, as a consequence of constant interaction with multiple environmental agents resulting in tissue injury and aberrant repair [4]. It has an important role in the origin and progression of asthma as well as in exacerbations. 

According to Holgate, in asthma, the epithelial-mesenchymal trophic unit (EMTU), which controls the local tissue microenvironment and maintains tissue homeostasis, becomes deregulated [5]. On the basis of this concept, epithelial damage by environmental agents such as biologically active allergens, air pollutants, irritants, environmental tobacco smoke, and respiratory viruses, results in the production of signals that act on the underlying mesenchyme to propagate and amplify inflammatory and remodelling responses in the submucosa. This concept is further strengthened by the fact that many asthma susceptibility genes identified so far are expressed in the epithelium and mesenchyme. 

There is also a strong evidence base supporting the proposition that IgE-mediated mechanisms play a crucial role in asthma pathogenesis. IgE synthesis may take place in the bronchial mucosal epithelium of patients with both atopic and nonatopic asthma [6].

#### 2.1.1. Atopic and Nonatopic Asthma: The Concept

Rackemann first introduced the terms “extrinsic” and “intrinsic” asthma in 1947 [7]. The author observed in a study of a limited number of patients a form of the disease in which extrinsic factors were triggering the symptoms and another in which no specific triggering factor could be found. He called the former “extrinsic asthma” and the latter “intrinsic asthma”. According to Rackemann, the first type was easier to diagnose, associated with vasomotor rhinitis and allergy and of onset before the age of 30. The second type was more difficult to diagnose and associated with polypoid sinusitis. More recently, and not necessarily in line with Rackemann's view of external “triggers”, the differentiation between the two entities has been focussed on the presence or absence of positive skin prick (+/− serological) tests to common aero allergens rather than purely on the basis of history of allergies. A series of studies designed to analyse the immunopathological differences between atopic and nonatopic asthma resulted in the conclusion that both kinds of asthma share more similarities than differences [8, 9]. Nieves et al. conducted a relatively large population study in 751 asthmatics to understand the discriminating characteristics between the two groups and concluded that despite the immunopathological similarities, atopic and nonatopic asthma reflect distinct clinical phenotypes [10]. Age, age of onset of asthma, and female/male ratio were higher in nonatopic than in atopic asthmatics. Younger age, early onset, male sex, rhinitis, and smoking were independent factors discriminating atopic from nonatopic asthma. In the above survey, about a quarter of patients were nonatopic, whereas an earlier Swiss SAPALDIA survey found that a third of total asthmatics were nonallergic (intrinsic) [11, 12].

Ever since the first description of intrinsic asthma, there has been debate about its pathogenesis [11]. It has been suggested that nonatopic asthma may represent a form of autoimmunity possibly triggered by a viral infection like influenza which often precedes its onset. This is further supported by the recent observation that nonatopic asthmatics frequently demonstrate positive autologous serum skin tests, evidence of circulating histamine releasing factors and positive ANA autoantibodies [13]. It is also always possible that “nonatopic” asthmatics manufacture IgE against as yet undetected or unidentified “allergen” and, therefore, may benefit from allergen avoidance [14, 15].

The airway epithelium in atopic or nonatopic asthma may sometimes be colonized by microbes such as staphylococci which produce superantigens. Superantigens produced locally in the airways may lead to class switching of local B-cells, resulting in polyclonal IgE production in the airways and also the production-specific IgE against the super antigens [16, 17]. This leads to sensitization of mast cells, which can be activated by the usual asthma triggers such as exercise. Superantigens can also stimulate clonal expansion of T-cells, resulting in increased Th2 cells and CD8^+^ cells while suppressing regulatory T cells [18, 19].

#### 2.1.2. Airway Epithelium in Nonatopic and Atopic Asthma: Similarities or Differences?

Asthma is characterised by the presence of bronchial mucosal infiltrate with eosinophils and elevated expression of eosinophil active chemokines and cytokines, indicating local T-cell activation. Early comparisons of epithelial cellular infiltrates in atopic and nonatopic asthma showed remarkable similarities, except for increased tissue macrophages in the bronchial mucosa of the nonatopic patients [20, 21]. This is linked to the fact that bronchoalveolar lavage cells expressing the *α*-subunit of the granulocyte macrophage colony stimulating factor receptor (GM-CSFr), expressed by alveolar macrophages, were increased in nonatopic but not in atopic asthma [22].

The majority of the studies comparing atopic and nonatopic asthma have been focussed on the inflammatory and/or immune cells at the submucosal level rather than the resident cells in the mucosal epithelium. In this paper, we bring together what few comparisons have been made between atopic and nonatopic asthma at the epithelial and submucosal level. 

Shahana and colleagues compared the ultrastructural changes in the airway epithelium in bronchial biopsies using transmission electron microscopy in a relatively small group of patients with allergic (*n* = 11) and nonallergic (*n* = 7) asthma [23]. The patients were matched for disease severity, peak flow, and bronchial hyperreactivity. Interesting similarities and differences between the two groups were noted from the results of this study and another study by Amin et al. [24], as discussed below (Table 1).

**Epithelium**

Epithelial damage mainly affecting columnar cells was found to be extensive in both allergic and nonallergic asthmatics compared to controls in the electron microscopic study using a semiquantitative scoring system. The damage was slightly higher in atopics; however, the difference was not significant. The columnar cells had been lost over larger areas in severe cases, with only few basal cells left covering the basement membrane in extreme cases. Areas with flattened rather than columnar cells were observed only in nonallergic asthma [23]. By contrast, the degree of epithelial damage was significantly higher in the atopic asthma group in the study by Amin et al. [24]. In the damaged areas, cylindrical ciliated epithelial cells were absent, whereas the layer of cuboidal basal cells was often intact. Differences in the criteria for defining epithelial damage and the techniques used (transmission electron microscopy versus immunohistochemistry) may explain these disparities.

**Desmosomes**

Desmosomes are specialized major cell-cell adhesive junctions of epithelial tissue and play an important role in maintaining tissue integrity via intermediate filaments. They also play a role in the transduction of intracellular signals that regulate cell behaviour [25, 26]. In the bronchial epithelium, desmosomes were present between columnar cells, columnar and basal cells, and between basal cells. The relative length of the desmosomes was reduced in both types of asthma, more so in the columnar cells in allergic asthma. Half desmosomes were also common in this group consisting of a single plaque in one of the adjacent cells [23]. The authors speculated that asthma patients have an intrinsic or acquired deficiency in the synthesis of desmosomes, which would make the epithelium more prone to loss of columnar cells.

**Basal Cells**

In the basal cells, the relative desmosomal lengths were significantly reduced both in allergic and nonallergic asthma compared to controls [23]. Basal cells were also lost in asthma, however, less so compared with columnar cells, probably due to the presence of stronger desmosomes in basal cells.

**Goblet Cells**

Goblet cell hyperplasia, partly responsible for increased mucus secretion, was observed only in allergic asthmatics. As mast cell chymase is one stimulus for mucus secretion, increased mast cell degranulation in allergic asthma may at least partly account for this. This finding was repeated by others [27].

**Basal Lamina**

The subepithelial basement membrane in human airways is composed of a basal lamina and reticular basement membrane. Subepithelial fibrosis is thought to cause basement membrane thickening in asthma. Under the electron microscope, thickening of lamina densa of the basal lamina was more prominent in allergic asthma [23]. Many other studies describe an increase in the thickness of basement membrane in asthmatics, more so in atopic asthma, on the basis of the light microscopic immunohistochemical examination of the bronchial mucosa [28–30]. The tenascin and laminin layers were significantly thicker in atopic asthma compared to nonatopics [24]. The significance of these changes is still not clear.

#### 2.1.3. Inflammatory Cells

**Eosinophils**

In asthmatics, many studies show a correlation between eosinophilic infiltration and epithelial damage [31, 32]. Studies comparing the numbers of mucosal epithelial eosinophils in atopic and nonatopic asthmatics [20, 24, 33] show disparate findings. Some studies noted a significant increase in eosinophil numbers in the atopic compared to nonatopic asthmatics by immunohistochemistry (EG1 and EG2 positivity) [24]. Activated eosinophils were also more numerous in atopic asthmatics. On the other hand, other studies reported that eosinophils were more abundant in the bronchial submucosa of nonatopic than atopic asthmatics for a given degree of disease severity [20, 33].

**Neutrophils**

One study reported elevated airways epithelial neutrophils (HNL- and MPO-positive cells) in nonatopic asthmatics compared to atopics and nonasthmatic control groups [24]. Increased neutrophilic infiltration of the airways [34] has been reported in some studies to be a more generalised feature of severe asthma (an observation which may also reflect the effects of corticosteroid therapy).

**Mast Cells**

Mast cell numbers appear to be increased in the airways in atopic and nonatopic asthma and found to be similarly increased in both groups by immunohistochemical staining of antitryptase antibody-1(AA1). In one study, the total number and the number of degranulating mast cells were higher in allergic asthmatics by electron microscopy though the difference was not statistically significant [23]. However, there lack evidence showing differences in infiltrated inflammatory cells in epithelium between atopic and nonatopic asthma.

**Lymphocytes**

Profuse lymphocyte infiltration was observed equally in both atopic and nonatopic asthmatics by electron microscopy [23]. Other studies are disparate, with some suggesting intense mononuclear cell infiltration with an increase in the number of CD45^+^ cells (total leukocytes), CD3^+^, and CD4^+^ T lymphocytes in nonatopic asthmatics [21], but others showing elevated total numbers T-lymphocyte of all subsets (CD3, CD4, CD8, and CD25) in the bronchial epithelium of atopic as compared with nonatopic asthmatics by immunohistochemical staining [24].

**Macrophages**

Some studies have shown an increased number of CD68^+^ tissue macrophages in the bronchial mucosa of nonatopic, as compared with atopic asthmatics [20, 21]. There was an increased number of cells expressing the *α*-subunit of the GMCSF receptor, reflecting the elevated number of these tissue macrophages [22].

## 3. Cytokines and Their Receptors

Atopic and nonatopic asthmatics had higher numbers of cells expressing IL-4, IL-5 and IL-8 than control subjects according to Amin et al. [24]. The numbers of cells expressing IL-4 and IL-5 were higher in atopic asthmatics, whereas IL-8 expression was higher in bronchial epithelial cells and neutrophils in nonatopic asthmatics by immunohistochemistry. On the other hand, studies by our group suggested that IL-4 and IL-5 protein positive cells and m-RNA were equally and significantly elevated in both atopic and nonatopic asthmatics compared to controls [8]. IL-4 and IL-5 receptors (IL-4R*α* and IL-5R*α*) expression was also equally elevated in the two groups. There are also reports of elevated numbers of epithelial cells and eosinophils expressing IL-8 in atopic asthma [35, 36]. Two separate studies reported marked increase in CD68^+^ macrophages in nonatopic, compared with atopic asthma as shown by an increase in CD68^+^ [20, 33] or in GMCSF receptor *α* subunit^+^ cells [22]. The significance of this finding remains unclear. However, according to the observations made by another group, there were no differences in the amounts of mRNA encoding GMCSF, IL-3, or IL-13 by in situ hybridization between the two groups [37]. Concentrations of IL-2 and *γ*-IFN were observed to be elevated in the BAL or sputum fluid of nonatopic compared to atopic asthmatics; however, IL-2 receptors were equally expressed in both groups [38, 39]. The expression of IL-10 and IL-12 m-RNA was increased in the sputum of atopic asthmatics compared to nonatopics, suggesting the presence of a homeostatic mechanism to reduce lung inflammation [40] (Table 2). 

## 4. Chemokines and Receptors

According to multiple studies, both atopic and nonatopic asthmatics showed enhanced expression of the CC chemokines eotaxin, eotaxin-2, RANTES, monocyte chemotactic protein 3 (MCP-3), and MCP-4 in the bronchial epithelium and submucosa with no differences between them [37, 41, 42]. In one study, the mean concentration of RANTES in BAL cell supernatants was increased in nonatopic asthmatics compared to controls but very low in atopic asthmatics [43]. Many C-C chemokines are potent eosinophil chemoattractants and act predominantly via the CCR3 receptor, the expression of which was also similar in the two groups, either in epithelium or submucosa (Table 3).

## 5. B Cells and IgE Mechanisms

The role of IgE in asthma pathogenesis is likely complex and not fully understood. The Th2 cytokines IL-4 and IL-13, overproduced in asthma, are the only known cytokines which can induce B cell switching to IgE synthesis, but precisely where and when such switching occurs is unclear. In addition to mediating acute degranulation of mast cells and basophils through its binding to Fc*ε*RI, which may at least exacerbate asthma in some individuals, IgE may play important, less well-defined roles in the genesis of asthmatic mucosal inflammation, for example, by facilitating antigen capture and presentation by subjects of mucosal dendritic cells expressing Fc*ε*RI and B cells themselves which express Fc*ε*RII (CD23). These mechanisms may operate regardless of whether allergen-specific IgE in detectable in the periphery (by skin-prick testing or in vitro tests on the serum). Furthermore, IgE responses in asthmatics might not be restricted to conventional aeroallergens. Epidemiologically, individuals who overproduce IgE (of any specificity) are more likely to develop asthma, but this does not necessary indicate cause and effect. Perhaps the clearest evidence for a role for IgE in asthma pathogenesis comes from the therapeutic success of anti-IgE strategies in asthma. Omalizumab, for example, which prevents IgE binding to Fc*ε*RI and Fc*ε*RII (CD23), reduces asthma exacerbations and mucosal inflammation at least in atopic asthmatics, and its possible effects in nonatopic asthma remain to be seen. 

In a series of bronchial biopsy studies using in situ hybridization and/or immunohistochemistry, both atopic and nonatopic asthma were characterised by increased infiltration of Th2 cells secreting IL-4, the presence of Fc*ε*RII^+^ cells and cells that expressed mRNA for the *ε* germ-line transcript (I*ε*), and *ε* heavy chain of IgE (C*ε*) compared to nonasthmatic controls (Table 4) [44]. Interestingly, there were no differences in the numbers of CD20^+^ B-cells between asthmatics (atopic and nonatopic) and nonasthmatic controls. These observations combined with that of upregulation of Fc*ε*R1 expression on mast cells, macrophages, and eosinophils in the epithelial submucosa of asthmatics irrespective of their atopic status support the notion that localised IgE synthesis and class switching take place in the asthmatic bronchial epithelium regardless of the conventional atopic status of the individual [33]. In another study from our group, *ε*-GLT and AID mRNA were detectable in the bronchial mucosa of asthmatics and nonasthmatic controls, by RT-PCR; however, circle transcripts (I*ε*-C*μ*, I*ε*-*γ*) and *ε*-mRNA were detectable mainly in asthmatics, but rarely in nonasthmatics [45]. These findings further strengthen our hypothesis that the asthmatic epithelium is primed for localised IgE switching and IgE synthesis, irrespective of the subject's atopic status.

## 6. Conclusion

Even though subtle differences exist between atopic and nonatopic asthma at the mucosal and submucosal levels, the fact is that the similarities invariably outweigh the differences. Having said this, many aspects of the pathogenesis of nonatopic asthma still remain incompletely resolved, in particular the role of IgE. Further well-designed experiments are necessary to address these questions. 

## Figures and Tables

**Table 1:** Comparison of bronchial epithelial components in atopic and nonatopic asthma.

Cell types or epithelial components	Atopic asthma	Nonatopic asthma
Ciliated columnar	Damage ++	Damage + [23, 24]
Desmosomes	Breakdown ++	Breakdown + [23]
Goblet	Hyperplasia +	Hyperplasia − [23]
Basal cells	Damage +/−	Damage +/− [23]
Basement membrane	Thickening ++	Thickening + [23, 24, 28–30]
Eosinophils	Infiltration +++	Infiltration +++ [20, 24, 33]
Neutrophils	Infiltration +	Infiltration ++ [24, 34]
Mast cells	Infiltration ++	Infiltration + [23]
Lymphocytes	Infiltration +++	Infiltration ++ [21, 23, 24]
Macrophages	Infiltration +	Infiltration ++ [20–22]

**Table 2:** Cytokine and cytokine receptor expression in the respiratory epithelium and submucosa.

Cytokines and receptors		Atopic asthma	Nonatopic asthma
Th1	*γ* IFN (sputum)	++	++ [39]
	IL-2 (BAL fluid)	+	++ [38]
	IL-2R+	+	+ [38]

Th2	IL-4	++ +/−	++ [8]
	IL-4*α* receptor	++	++ [8]
	IL-5	++ +/−	++ [8]
	IL-5*α* receptor	++	++ [8]
	IL-10 (sputum)	++	+ [40]
	IL-13	++	++ [37]
	GMCSF	++	++ [37, 39]

Th1 & Th2	IL3	++	++ [37]

Macrophage	GMCSFR-*α*	+	+++ [22]

**Table 3:** Chemokine and chemokine receptor expression by the respiratory epithelium and submucosa.

Chemokine and/or (receptor)	Atopic asthma	Nonatopic asthma
IL-8	++	++ +/− [24, 35, 36, 43]
IP10(CXCR3)	++ [46]	?
I-TAC(CXCR3)	+/− [46]	?
RANTES(CCR 3)	++	+++/− [37, 41–43]
MCP-1(CCR2/4)	++	++ [43]
MCP-3 & 4(CCR3)	++	++ [37, 41, 42]
Eotaxin(CCR3)	++	++ [37, 41, 42]
TARC(CCR4)	++ [46, 47]	?
MDC(CCR4)	++ [46, 47]	?
CCR8	+ [47]	?

?—Comparison/data not available.

**Table 4:** IgE and related molecules in the respiratory mucosa [42, 47].

IgE-related expression	Atopic asthma	Nonatopic asthma
CD20^+^ B cells	++	++ [44]
Fc*ε*RI^+^ cells	++	++ [44]
*ε*-GLT	+++	+++ [45]
I*ε*-C*μ*CT	++	++ [44, 45]
I*ε*-C*γ*CT	++	++ [45]
*ε*-mRNA	+++	+++ [45]
AID-mRNA	++	++ [45]
